# Troponin I is an independent marker of cardiovascular mortality risk in chronic kidney disease patients

**DOI:** 10.1590/2175-8239-JBN-2025-0013en

**Published:** 2025-09-19

**Authors:** Gabriela Romaniello, Shirley Yumi Hayashi, Bruno Siegel Guerra, Miguel Carlos Riella, Bengt Lindholm, Gustavo Lenci Marques, Marcelo Mazza do Nascimento

**Affiliations:** 1 Universidade Federal do Paraná, Hospital de Clínicas, Departamento de Medicina Interna, Curitiba, PR, Brazil.; 2 Karolinska University Hospital, Karolinska Institute, Department of Clinical Science, Intervention and Technology, Divisions of Renal Medicine and Baxter Novum, Huddinge, Stockholm, Sweden.; 3 Fundação Pró-Renal, Departamento de Nefrologia, Curitiba, PR, Brazil.; 4 Pontifícia Universidade Católica do Paraná, Departamento de Biotecnologia, Curitiba, PR, Brazil.; 5 Hospital Evangélico de Curitiba, Departamento de Medicina Interna, Curitiba, PR, Brazil.; 6 Munai, Curitiba, PR, Brazil.

**Keywords:** Troponin, Renal Insufficiency, Chronic, Cardiovascular Diseases, Cardiac Events

## Abstract

**Introduction:**

Cardiovascular disease (CVD) is the main cause of death among chronic kidney disease (CKD) patients. However, the cardiovascular (CV) prognostic evaluation in CKD is not established. Despite previous reports establishing troponin as a CV mortality prognosticator in CKD, there is no consensus on its applicability. Moreover, studies on high-sensitivity troponin I (hsTnI) in this context are scarce. We evaluated the association between hsTnI and CV and overall mortality among CKD patients to identify higher CV-risk patients.

**Methods:**

145 patients with CKD stages 3 to 5 underwent measurements of hsTnI, inflammatory, calcium-phosphorus metabolism, vascular calcification, and echocardiographic parameters. The association of hsTnI with CV and overall mortality after follow-up was established using Kaplan-Meier curves. The cutoff value of hsTnI for predicting CV and overall mortality was defined using ROC curve analysis. Multivariate analysis for CV and overall mortality was done using Cox regression models.

**Results:**

HsTnI cutoff value for overall and CV mortality was 0.057 ng/mL. Patients with higher hsTnI had higher CV and overall mortality. In multivariate analysis, hsTnI was a marker of increased CV mortality (hazard ratio 12.8 (95% CI 1.56–105.08), p = 0.018), independent of age, sex, previous CVD, diabetes and dialysis, echocardiographic findings, and osteo­protegerin (OPG).

**Conclusion:**

HsTnI is independently associated with CV mortality in CKD patients, suggesting that it may be a potential CV risk stratification marker.

## Introduction

CVD is the leading cause of death among CKD patients^1^. The high prevalence of established risk factors such as hypertension, diabetes, and dyslipidemia in CKD patients does not fully explain their exponentially elevated CV mortality^2,3^ Hence, novel markers have been studied over the past years to predict CV risk in CKD^2,4, 5, 6^.

Troponin T (TnT) and troponin I (TnI) are cardiac biomarkers mostly used to diagnose acute myocardial infarction^5^. However, their role as prognostic markers in CKD has been increasingly recognized in recent years^7,8^. Although both markers reflect myocardial injury, TnI may offer greater specificity than TnT in this context^5,7,8^. This occurs mostly due to differences in their molecular structure, expression patterns, and clearance mechanisms. High-sensitivity troponin T (hsTnT) is more frequently elevated in CKD patients, even in the absence of cardiovascular disease, probably due to its potential skeletal muscle expression, association with inflammation, and increased impact from reduced renal clearance. In contrast, high-sensitivity troponin I (hsTnI) demonstrated to be less influenced by non-cardiac factors and may be more accurate to indicate cardiac injury, particularly in patients with impaired renal function. These differences highlight the potential advantage of hsTnI as a more specific and clinically useful prognostic biomarker in CKD population, as demonstrated in recent studies^9^.

This study aimed to investigate the prognostic impact of hsTnI on overall and CV mortality compared to other inflammatory, vascular calcification, calcium-phosphorus metabolism, and echocardiographic markers in a cohort of CKD patients stages 3 to 5.

## Methods

### Patients

Patients with CKD stages 3–5, including those undergoing dialysis at Pró-Renal Foundation in Brazil, were considered for enrollment. All patients gave their written informed consent, and the Hospital Evangélico de Curitiba ethics committee approved the study protocol, under number 0537/08 on January 30, 2008, following the established procedures at the time^10^. The exclusion criteria were dialysis treatment lasting less than 1 month, age younger than 18 years, presence of HIV or hepatitis B/C infection, and other chronic inflammatory diseases.

All patients enrolled underwent a baseline investigation comprising blood sampling and CV assessment. They were subsequently followed up for survival analysis. The observation period was from March 2008 to December 2015.

### Cardiovascular Assessment and Laboratory Analyses

All patients underwent echocardiography to establish ejection fraction (EF), left ventricular mass index (LVMI), and diastolic dysfunction (DD)^10^. Blood samples were collected from patients after overnight fasting. The samples were collected midweek from hemodialysis patients and at regular clinic visits in other patients with CKD stages 3–5, including those undergoing peritoneal dialysis. Laboratory analysis included cardiac markers (hsTnI), inflammation-nutritional markers (C-reactive protein [CRP], tumor necrosis factor alfa [TNFalfa], interleukin 6 [IL-6], pentraxin 3 [PTX3], albumin), calcium-phosphorus metabolism marker (fetuin, fibroblast growth factor-23 [FGF23]), vascular calcification marker (OPG), oxidative stress markers (S100 calcium-binding protein A [S100A], receptor for advanced glycation end products soluble form [sRAGE]), and hemoglobin levels. All analyses were performed using automated analyzers at the Renal Medicine Laboratory, Clinical Research Center, Karolinska Institutet, Stockholm, Sweden. Serum hsTnI was measured with an immunometric assay by the Immulite 1000 Analyzer (Siemens Medical Solutions Diagnostics).

### Statistical Analysis

Data are reported as median (minimum and maximum) or mean ± standard deviation (SD) and as frequencies and percentages for categorical variables, as appropriate. For determining the hsTnI cutoff value associated with CV and overall mortality, receiver operating characteristic (ROC) curves were adjusted and the corresponding area under curve (AUC) was evaluated. The best cutoff was determined using the Youden index criteria. To compare two groups with continuous quantitative variables, we used Student’s t-test for independent samples. The Mann-Whitney U test was applied for the non-normally distributed variables. To verify the association between two qualitative variables, chi-square test was used, and Fisher test was added when necessary. Additionally, a univariate analysis was performed estimating Spearman’s correlation coefficients.

Univariate analysis was performed using Cox regression to analyze factors associated with CV and overall mortality, and the results were expressed as hazard ratio (HR). The following variables were included in the univariate analysis: hs-TnI, male sex, DM, OPG, dialysis, DD, age, Hb, TNFalfa, LVMI, EF, CRP, fetuin, sRAGE, PTX3, FGF23, IL-6, and S100A. Subsequently, multivariate regression models also using Cox were performed, including clinically relevant variables. The first multivariate analysis model comprised hsTnI and demographic and clinical variables. The second model used the same variables as the first one plus echocardiographic parameters. The third model added OPG. The estimated association measure was HR provided for unit change with a 95% confidence interval (CI). Kaplan-Meier curves were used to analyze survival. The normality of the variables was evaluated using the Shapiro-Wilk test. P values < 0.05 were considered statistically signifi­cant. Data were analyzed using JAMOVI v. 2.5.0.

## Results

### Baseline Characteristics

We included 145 (89 males) patients (54 non-dialysis, 36 hemodialysis (HD), and 55 peritoneal dialysis). The median follow-up was 36.5 months. The number of deaths was 56, and specific causes included CV (25), infection (17), malignancies (4), and other causes (10). Clinical and biochemical characteristics of the patients based on troponin levels and survival status are summarized in Tables 1 and 2, respectively. The median hsTnI cohort levels was 0.0703 ng/mL [0.00181, 1.68].

**Table 1:** Clinical and biochemical characteristics according to hsTnI as defined by its cutoff value, 0.057 ng/ml, for predicting overall and cv mortality

	Higher hsTnI ≥ 0.057ng/mLn = 58	Lower hsTnI < 0.057ng/mLn = 87	P-value
Age (years)	62 (18;87)	58 (24;92)	0.176
Sex (male)	49 (59.0%)	40 (66.7%)	0.451
Diabetes (yes)	31 (37.3%)	11 (18.3%)	0.0227
Dialysis (yes)	65 (78.3%)	26 (43.3%)	0.001
Hb (g/dL)	11.2 (6;18.5)	12.5 (6.7;18.8)	0.0599
hs-CRP (mg/dL)	5.9 (0.330;74.9)	2.6 (0.3;69.1)	0.0237
IL-6 (pg/mL)	5.9 (0.330;74.9)	2.6 (0.3;69.1)	0.0546
OPG (pmol/L)	10.8 (3.72;33.2)	7.07 (1.89;18.9)	0.001
Fetuin (pg/mL)	0.418 (0.105)	0.419 (0.0984)	0.948
TNF-α (pg/mL)	18.1 (7.1;56.2)	14.5 (7.3;43.5)	0.0447
PTX3 (pg/mL)	4.12 (1.33;94.6)	3.69 (1.48;18.8)	0.32
FGF-23 (pg/mL)	507 (60;79600)	186 (44;40100)	0.15
s-RAGE (pg/mL)	2320 (540;5180)	1660 (472;5780)	0.014
S100A (ng/mL)	45.1 (5.02;464)	52.5 (6.08;160)	0.01
EF%	63 (27;84)	70 (48;88)	0.001
Diastolic dysfunction	62 (74.7)	35 (58.3)	0.0593
LV mass index (g/m^2^)	62 (30.2;160)	51.5 (30.6;108)	0.00257
Troponin-I (ng/mL)	0.0322 (0.0151)	0.1811 (0.2478)	–
All cause death	44 (53.0%)	12 (20.0%)	0.001
CV death	25 (30.1%)	2 (3.3%)	0.001

Abbreviations – Hb: hemoglobin; hsCRP: high-sensitivity C-reactive protein; IL-6: interleukin-6; OPG: osteoprotegerin; TNF-α: tumor necrosis factor alpha; PTX3: pentraxin 3; FGF23: fibroblast growth factor-23; sRAGE: receptor for advanced glycation end products soluble form; S100A: S100 calcium-binding protein A; EF: ejection fraction; LV: left ventricle; CV: cardiovascular. Notes – Quantitative normal variables are expressed as mean (standard deviation). Quantitative non-normal variables are expressed as median (minimum, maximum). Categorical variables are expressed as frequencies (percent).

**Table 2:** Clinical and biochemical characteristics according to survival status

	Survivors n = 89	Non-survivors n = 56	P-value
Age (years)	58 (16;83)	64 (25;92)	0.00198
Sex (male)	59 (66.3%)	30 (53.6%)	0.175
Diabetes (yes)	26 (29.2%)	17 (30.4%)	1
Dialysis (yes)	42 (47.2%)	49 (87.5%)	0.001
Hb (g/dL)	12.4 (7.8;18.8)	10.9 (6;18.5)	0.011
hs-CRP (mg/dL)	3.2 (0.3;54.4)	5.8 (0.660;74.9)	0.128
IL-6 (pg/mL)	3.9 (0.09;98.7)	4.9 (0.1;95.1)	0.249
OPG (pmol/L)	7.56 (1.89;18.9)	12.3 (2.37;33.2)	0.004
Fetuin (pmol/L)	0.428 (0.101)	0.407 (0.103)	0.238
TNF-α (pmol/L)	16.8 (1.5;56.2)	17.9 (7.3;34.5)	0.611
PTX3 (pg/mL)	3.83 (1.46;94.6)	4.02 (1.33;18.8)	0.17
FGF-23 (pg/mL)	240 (44;63700)	461 (61;79600)	0.69
s-RAGE (pg/mL)	1770 (540;5780)	2230 (472;5170)	0.091
S100A (ng/mL)	42.9 (6.08;464)	65.1 (5.02;409)	0.2
Troponin-I (ng/mL)	0.0521 (0.00181;0.488)	0.105 (0.0212;1.68)	0.00715
EF%	67 (31;88)	63 (27;82)	0.0654
Diastolic dysfunction	55 (61.8%)	44 (78.6%)	0.0536
LV mass index (g/m^2^)	57.4 (30.2;115)	62.1 (40.6;160)	0.0826

Abbreviations – Hb: hemoglobin; hsCRP: high-sensitivity C-reactive protein; IL-6: interleukin-6; OPG: osteoprotegerin; TNF-α: tumor necrosis factor alpha; PTX3: pentraxin 3; FGF23: fibroblast growth factor-23; sRAGE: receptor for advanced glycation end products soluble form; S100A: S100 calcium-binding protein A; EF: ejection fraction; LV: left ventricle; CV: cardiovascular. Notes – Quantitative normal variables are expressed as mean (standard deviation). Quantitative non-normal variables are expressed as median (minimum, maximum). Categorical variables are expressed as frequencies (percent).

Univariate correlations using Spearman’s rank, demonstrated that hsTnI levels were significantly associated with Hb (r = -0.197, p = 0.024), CRP (r = 0.338, p < 0.001), IL-6 (r = 0.421, p < 0.001), OPG (r = 0.544, p < 0.001), TNFα (r = 0.269, p < 0.001), EF (r = -0.385, p < 0.001) and LVMI (r = 0.298, p < 0.001).

### Troponin Levels and Clinical Outcomes

**Figure 1:**
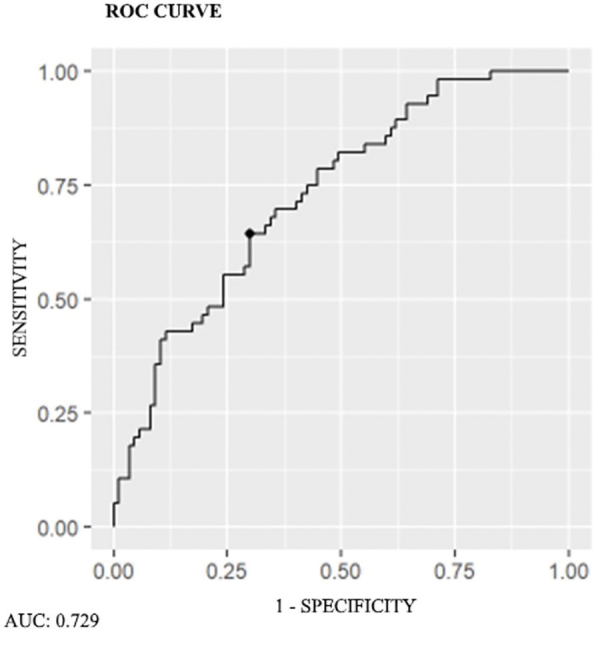
ROC curve for CV and overall mortality among 145 patients with CKD stages 3–5. For hsTnI of 0.057 ng/mL (cutoff value), the area under the curve [AUC] was 0.729 [0.669–0.779].

The hsTnI cutoff value was determined based on the ROC curve for general and CV mortality and was 0.057 ng/mL for both outcomes (AUC 0.729 [0.669–0.779]) (Figure 1). Univariate analysis of mortality risk showed that elevated hsTnI levels (>0.057 ng/mL) were related to higher overall and CV mortality (HR 4.68; 95% CI: 2.05–10.69, p < 0.001 and HR 19.18; 95% CI: 2.56–143.76, p = 0.004, respectively) as were OPG levels (HR 5.87, 95% CI: 2.83–12.21, p < 0.001 and HR 4.63, 95%CI: 1.77–12.06), p = 0.002, respectively) and dialysis treatment (HR 6.31, 95% CI: 2.45–16.30, p < 0.001 and HR 8.24, 95% CI: 1.90–35.73, p = 0.005, respectively).

**Figure 2:**
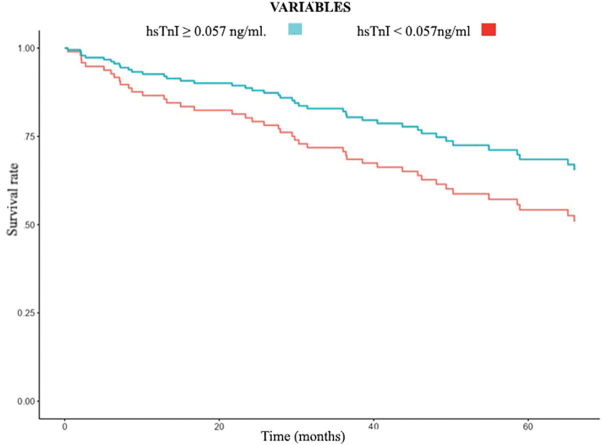
Kaplan Meier curve for overall mortality according to baseline hsTnI levels in CKD patients. Red curve represents hsTnI < 0.057 ng/mL and blue curve represents hsTnI ≥ 0.057 ng/mL.

**Figure 3:**
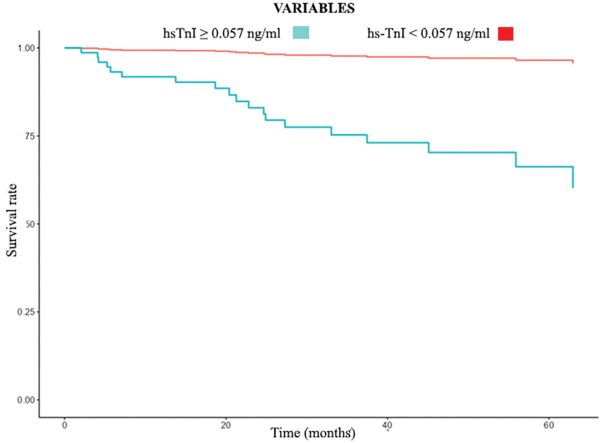
Kaplan Meier curve for CV mortality according to baseline hsTnI levels in CKD patients. Red curve represents hsTnI < 0.057 ng/mL and blue curve represents hsTnI ≥ 0.057 ng/mL.

Kaplan-Meier cumulative incidence curves showed no difference in overall mortality (Figure 2) but higher CV mortality (Figure 3) in the higher hsTnI group (>0.057 ng/mL) early in the follow-up period, with an increase in the gap over time. In multivariate analysis (Table 3), in a model including age, male sex, diabetes, previous CV disease, and dialysis, hsTnI was an independent marker of CV mortality. The result remained statistically significant when DD, LVMI, and EF were added to the analysis. When OPG was included in the study, hsTnI persisted as an independent CV mortality marker (HR 12.8 (95%CI: 1.56–105.08), p = 0.018). Regarding overall mortality, in the multivariate analysis model, including age, male sex, diabetes, previous CV disease, and dialysis, troponin was an independent marker. However, when DD, LVMI, EF, and OPG were added to the analysis, troponin was no longer an independent overall mortality marker (Table 4).

**Table 3:** Multivariate analysis of variables associated with cv mortality risk

Models	Variable	HR	95%CI	p value
Model-1	hsTnI (≥ 0.057ng/mL)	6.62	1.44–30.37	0.015
	Age (yes)	1.01	0.97–1.05	0.621
	Male (yes)	0.96	0.40–2.30	0.922
	DM (yes)	0.68	0.26–1.79	0.436
	CVD (yes)	1	0.37–2.76	0.995
	dialysis (yes)	4.88	1.08–22.17	0.04
Model-2	hsTnI (≥ 0.057ng/mL)	12.39	1.51–102	0.019
	Age (years)	1.01	0.97–1.06	0.565
	Male (yes)	1.04	0.37–2.95	0.943
	DM (yes)	0.53	0.16–1.70	0.284
	dialysis (yes)	3.96	0.83–19.00	0.085
	diastolic dysfunction (yes)	0.92	0.33–2.58	0.873
	CVD (yes)	0.76	0.22–2.68	0.674
	EF (%)	1	0.95–1.04	0.897
	LV mass index (g/m^2^)	1.01	0.99–1.04	0.342
Model-3	hsTnI (≥ 0.057 ng/mL)	12.8	1.56–105.08	0.018
	Age (years)	1.02	0.97–1.07	0.490
	Male (yes)	1.09	0.38–3.16	0.847
	DM (yes)	0.53	0.16–1.70	0.284
	Dialysis (yes)	4.30	0.77–23.90	0.097
	diastolic dysfunction (yes)	0.94	0,31–2.81	0.912
	EF (%)	1.01	0.95–1.04	0.789
	LV mass index (g/m^2^)	1.01	0.99–1.04	0.290
	OPG (pmol/L)	0.99	0.91–1.09	0.881
	CVD (yes)	0.86	0.23–3.21	0.817

Abbreviations – OPG: osteoprotegerin; CVD: cardiovascular disease; CKD: chronic kidney disease; hsTnI: high sensitivity tronponin I; HR: hazard ratio; 95% CI: 95% confidence interval.

**Table 4:** Multivariate analysis of variables associated with all-cause mortality risk

Models	Variable	HR	95% CI	p value
Model-1	hsTnI (≥ 0.057ng/ml)	2.15	1.02–4.54	0.044
	Age (years)	1.03	1.01–1.05	0.046
	Male (yes)	0.64	0.36–1.15	0.135
	CVD (yes)	0.97	0.48–1.99	0.944
	DM (yes)	0.81	0.41–1.57	0.529
	dialysis (yes)	4.16	1.76–9.83	0.001
Model-2	hsTnI (≥ 0.057 ng/mL)	1.97	0.81–4.77	0.133
	Age (years)	1.03	1.00–1.06	0.098
	Male (yes)	0.58	0.30–1.14	0.115
	DM (yes)	0.77	0.37–1.61	0.489
	CVD (yes)	0.78	0.35–1.75	0.544
	dialysis (yes)	4.11	1.65–10.20	0.002
	diastolic dysfunction (yes)	1.42	0.62–3.25	0.413
	EF (%)	0.95	0.93–1.01	0.247
	LV mass index (g/m^2^)	1.01	0.99–1.02	0.533
Model-3	hsTnI (≥ 0.057 ng/mL)	1.96	0.81–4.77	0.138
	Age (years)	1.03	0.99–1.06	0.124
	Male (yes)	0.58	0.29–1.14	0.114
	DM (yes)	0.77	0.37–1.61	0.489
	dialysis (yes)	4.01	1.44–11.16	0.008
	diastolic dysfunction (yes)	1.40	0.58–3.38	0.460
	LV mass index (g/m^2^)	1.01	0.99–1.02	0.545
	EF (%)	0.98	0.95–1.01	0.245
	OPG (pmol/L)	1.00	0.94–1.07	0.928
	CVD (yes)	0.78	0.34–1.77	0.556

Abbreviations – OPG: osteoprotegerin; CVD: cardiovascular disease; CKD: chronic kidney disease; hsTnI: high sensitivity tronponin I; HR: hazard ratio; 95% CI: 95% confidence interval.

## Discussion

HsTnI was shown to be an independent marker of CV mortality in patients with CKD, regardless of sex, age, inflammatory and oxidative markers, and other established CV risk factors including echocardiographic parameters and OPG. The exact mechanism that explains troponin elevation and its association with mortality is unknown^11^. It is hypothesized that subclinical coronary artery disease, supply versus demand imbalance related to cardiac hypertrophy, or direct myocardial injury might play a role in it^12^.

Our findings confirm higher CV mortality in CKD patients with increased hsTnI levels, regardless of dialysis therapy, demonstrating that hsTnI could be an independent CV mortality marker in this population. HsTnI might be more specific to indicate underlying heart disease and consequently CV mortality risk than TnT^13^. In a cohort of 20 thousand individuals comparing hsTnI and hsTnT measurements and clinical outcomes, Welsh et al.^9^ found that both markers had similar and robust associations with CV death but, after adjusting for established risk factors, only TnI was associated with myocardial infarction and chronic coronary disease. Similar to our study, TnI showed no association with non-CVD death. This finding is also in accordance with previous studies that suggested that TnT was more influenced by noncardiac diseases^14,15^.

Despite several studies reporting troponin association with CV mortality, uncertainty remains. Bargnoux found that CRP and brain natriuretic peptide were independent mortality predictors in HD patients, in contrast to TnI, which was found to have no association with overall mortality^16^. Satyan demonstrated a similar finding regarding TnT^17^. Our study did not find an association of CRP with CV or overall mortality neither in univariate nor in multivariate analysis, but a positive association of hsTnI with CV mortality, which might be explained by the high sensitivity assay. In contrast to our findings, a previous study from our group found a positive association of inflammatory markers with CV mortality in CKD patients^18^. A cohort study of CV mortality markers in CKD did not find associations with CRP and CV mortality in early stages of CKD and postulated that inflammation might mediate mortality only in advanced stages^19^. Since we included dialysis and non-dialysis patients, this might explain why inflammatory markers were not associated with mortality.

Our study is in accordance with a meta-analysis that evaluated TnT and TnI prognostic impact in CKD patients, nevertheless, few of the studies included performed multivariate analysis with adjusted data or used high sensitivity troponin assays^7^. This highlights the relevance of our study, which applied ROC curve analysis to determine a troponin cutoff value able to predict CV mortality and adjusted for variables including OPG, a marker of vascular calcification, inflammation, and a strong mortality predictor in CKD^20^.

CKD patients might present normal echocardiography and yet exhibit increased CV morbimortality^21^. However, cardiac remodeling is common and may determine worse prognosis^22^. Left ventricle hypertrophy, reduced EF, and DD are some of the echocardiographic signs classically related to poor prognosis in both general population and CKD patients^23, 24, 25, 26^. We found that increased LVMI and reduced EF were more frequent in patients with higher troponin levels, which is in accordance with the literature^27^.

Although echocardiographic alterations have been associated with increased mortality in CKD, previous studies did not include hsTnI in the multivariate analysis^28, 29, 30, 31^. Sun et al.^27^ evaluated hsTnT association with CV outcomes in HD patients and found that DD and LVMI had no association with CV and overall mortality when adjusted for hsTnT. We demonstrated similar findings, since hsTnI remained an independent marker of CV mortality independent of EF, LVMI, and DD.

The present study has several limitations that should be emphasized. First, its observational design restricts causal inferences between hsTnI levels and cardiovascular mortality. Second troponin analysis was based on one single sample, which restricts assessment of dynamic changes and their potential prognostic implications. Third, although the overall sample size was adequate for the primary analyses, it limited the power for detailed subgroup evaluations, particularly among dialysis versus non-dialysis patients and diabetic versus non-diabetic individuals. Future studies with larger sample sizes and serial biomarker measurements are required to validate and expand these findings.

In summary, the current study revealed that hsTnI is a strong prognostic indicator of CV mortality in CKD patients, whereas its association with overall mortality was weak. These findings suggest that TnI has a potential role in CV risk stratification in the CKD population. The identified cutoff value (0.057 ng/mL) could serve as a valuable clinical tool to identify high-risk patients who may benefit from intensified surveillance and early therapeutic interventions to reduce CV events. Incorporating hsTnI measurement into routine nephrology practice might facilitate personalized risk assessment, guiding clinicians in tailoring management strategies such as more aggressive control of traditional risk factors and prompt cardiology referral. Nonetheless, more studies are needed to better understand the mechanisms related to troponin elevation and its association with mortality, to evaluate hsTnI performance as part of prognostic risk models in combination with other biomarkers, and, finally, to investigate potential therapies to reduce the demonstrated CV risk.

## Data Availability

All data supporting the findings of this study are available from the corresponding author, Gabriela Romaniello, via e-mail (romaniello.gabriela@gmail.com) upon reasonable request. The dataset is not publicly available due to the presence of personally identifiable or sensitive health information, which could compromise participant confidentiality.
