# Supplementary material for: Troponin I is an independent marker of cardiovascular mortality risk in chronic kidney disease patients
Source: J Bras Nefrol. 2025 Sep 19;47(4):e20250013. doi: 10.1590/2175-8239-JBN-2025-0013en (PMC12459978; doi:10.1590/2175-8239-JBN-2025-0013en)
Supplement: Translation

# Translation: Troponina I como marcador independente de risco de mortalidade cardiovascular em pacientes com doença renal crônica

**DOI:** 10.1590/2175-8239-JBN-2025-0013pt

**Authors:** Gabriela Romaniello, Shirley Yumi Hayashi, Bruno Siegel Guerra, Miguel Carlos Riella, Bengt Lindholm, Gustavo Lenci Marques, Marcelo Mazza do Nascimento

**Affiliations:** 1 Universidade Federal do Paraná, Hospital de Clínicas, Departamento de Medicina Interna, Curitiba, PR, Brasil.; 2 Karolinska University Hospital, Karolinska Institute, Department of Clinical Science, Intervention and Technology, Divisions of Renal Medicine and Baxter Novum, Huddinge, Stockholm, Suécia.; 3 Fundação Pró-Renal, Departamento de Nefrologia, Curitiba, PR, Brasil.; 4 Pontifícia Universidade Católica do Paraná, Departamento de Biotecnologia, Curitiba, PR, Brasil.; 5 Hospital Evangélico de Curitiba, Departamento de Medicina Interna, Curitiba, PR, Brasil.; 6 Munai, Curitiba, PR, Brasil.

**Keywords:** Troponina de Alta Sensibilidade, Insuficiência Renal Crônica, Doenças Cardiovasculares, Mortalidade Cardiovascular, Mortalidade

## Abstract

**Introdução:**

Doença cardiovascular (DCV) é a principal causa de óbito entre pacientes com doença renal crônica (DRC). Entretanto, a avaliação prognóstica cardiovascular (CV) na DRC não está estabelecida. Apesar de relatos anteriores estabelecerem a troponina como prognóstico de mortalidade CV na DRC, não há consenso sobre sua aplicabilidade. Além disso, estudos sobre a troponina I de alta sensibilidade (hsTnI) nesse contexto são escassos. Avaliamos a associação entre hsTnI e mortalidade CV e geral entre pacientes com DRC para identificar pacientes com maior risco CV.

**Métodos:**

145 pacientes com DRC estágios 3 a 5 foram submetidos a medições de hsTnI, inflamação, metabolismo do cálcio-fósforo, calcificação vascular e parâmetros ecocardiográficos. A associação entre hsTnI e mortalidade CV e geral após o acompanhamento foi estabelecida por meio de curvas de Kaplan-Meier. O valor de corte da hsTnI para predição de mortalidade CV e geral foi definido com a análise da curva ROC. A análise multivariada para mortalidade CV e geral foi realizada utilizando modelos de regressão de Cox.

**Resultados:**

O valor de corte da hsTnI para mortalidade geral e CV foi 0,057 ng/mL. Pacientes com hsTnI mais elevada apresentaram maior mortalidade CV e geral. Na análise multivariada, a hsTnI foi um marcador de mortalidade CV aumentada (razão de risco 12,8 (IC 95% 1,56-105,08); p = 0,018), independente de idade, sexo, DCV anterior, diabetes e diálise, achados ecocardiográficos e osteoprotegerina (OPG).

**Conclusão:**

A hsTnI está independentemente associado à mortalidade CV em pacientes com DRC, sugerindo que pode ser um potencial marcador para estratificação de risco CV.

## Introdução

A DCV é a principal causa de óbito entre pacientes com DRC^1^. A alta prevalência de fatores de risco estabelecidos, como hipertensão, diabetes e dislipidemia em pacientes com DRC, não explica totalmente sua mortalidade CV exponencialmente elevada^2,3^ Por isso, novos marcadores têm sido estudados nos últimos anos para predizer o risco CV na DRC^2,4, 5, 6^.

A troponina T (TnT) e a troponina I (TnI) são biomarcadores cardíacos usados principalmente no diagnóstico de infarto agudo do miocárdio^5^. No entanto, seu papel como marcadores prognósticos na DRC tem sido cada vez mais reconhecido nos últimos anos^7,8^. Embora ambos os marcadores reflitam injúria miocárdica, a TnI pode oferecer maior especificidade do que a TnT nesse contexto^5,7,8^. Isso ocorre principalmente devido a diferenças em sua estrutura molecular, padrões de expressão e mecanismos de depuração. A troponina T de alta sensibilidade (hsTnT) encontra-se mais frequentemente elevada em pacientes com DRC, mesmo na ausência de doença cardiovascular, provavelmente devido à sua potencial expressão no músculo esquelético, associação com inflamação e maior impacto decorrente da redução da depuração renal. Em contrapartida, a troponina I de alta sensibilidade (hsTnI) demonstrou ser menos influenciada por fatores não cardíacos e pode ser mais precisa para indicar injúria miocárdica, especialmente em pacientes com função renal comprometida. Essas diferenças destacam a possível vantagem da hsTnI como um biomarcador prognóstico mais específico e clinicamente útil na população com DRC, conforme demonstrado em estudos recentes^9^.

O objetivo deste estudo foi investigar o impacto prognóstico da hsTnI na mortalidade geral e CV, em comparação com outros marcadores inflamatórios, de calcificação vascular, do metabolismo do cálcio-fósforo, bem como marcadores ecocardiográficos em uma coorte de pacientes com DRC nos estágios 3 a 5.

## Métodos

### Pacientes

Pacientes com DRC nos estágios 3-5, incluindo aqueles submetidos à diálise na Fundação Pró-Renal, Brasil, foram considerados para inclusão no estudo. Todos os pacientes forneceram seu consentimento informado por escrito, e o protocolo do estudo foi aprovado pelo Comitê de Ética do Hospital Evangélico de Curitiba, sob o número 0537/08, em 30 de janeiro de 2008, seguindo os procedimentos estabelecidos à época^10^. Os critérios de exclusão foram: tratamento dialítico com duração inferior a um mês, idade inferior a 18 anos, presença de infecção por HIV ou hepatite B/C e outras doenças inflamatórias crônicas.

Todos os pacientes incluídos foram submetidos a uma investigação basal que incluiu coleta de sangue e avaliação CV. Posteriormente, eles foram acompanhados para análise de sobrevida. O período de observação foi de março de 2008 a dezembro de 2015.

### Avaliação Cardiovascular e Análises Laboratoriais

Todos os pacientes foram submetidos à ecocardiografia para determinação da fração de ejeção (FE), do índice de massa do ventrículo esquerdo (IMVE) e da disfunção diastólica (DD)^10^. As amostras de sangue foram coletadas após jejum noturno. Para os pacientes em hemodiálise, as amostras foram coletadas no meio da semana. Para os demais pacientes com DRC nos estágios 3 a 5, inclusive aqueles em diálise peritoneal, as amostras foram coletadas durante visitas regulares à clínica. As análises laboratoriais incluíram marcadores cardíacos (hsTnI), marcadores inflamatórios relacionados à nutrição (proteína C reativa [PCR], fator de necrose tumoral alfa [TNF-alfa], interleucina 6 [IL-6], pentraxina 3 [PTX3], albumina), marcador de metabolismo do cálcio-fósforo (fetuína, fator de crescimento de fibroblastos-23 [FGF23]), marcador de calcificação vascular (OPG), marcadores de estresse oxidativo (proteína A de ligação ao cálcio S100 [S100A], forma solúvel do receptor para produtos finais de glicação avançada [sRAGE]) e níveis de hemoglobina. Todas as análises foram realizadas com analisadores automatizados no *Renal Medicine Laboratory*, *Clinical Research Center*, *Karolinska Institutet*, Estocolmo, Suécia. A hsTnI sérica foi medida por meio de um ensaio imunométrico pelo *Immulite 1000 Analyzer* (*Siemens Medical Solutions Diagnostics*).

### Análise Estatística

Os dados são apresentados como mediana (mínimo e máximo) ou média ± desvio padrão (DP) e como frequências e porcentagens para variáveis categóricas, conforme apropriado. Para determinar o valor de corte da hsTnI associado à mortalidade CV e geral, foram ajustadas as curvas ROC (*receiver operating characteristic*) e avaliada a área sob a curva (AUC) correspondente. O melhor ponto de corte foi determinado com base nos critérios do índice de Youden. Para a comparação de dois grupos com variáveis quantitativas contínuas, utilizamos o teste t de Student para amostras independentes. Para as variáveis com distribuição não normal, foi aplicado o teste U de Mann-Whitney. Para verificar a associação entre duas variáveis qualitativas, utilizou-se o teste do qui-quadrado, acrescido do teste de Fisher, quando necessário. Além disso, foi realizada uma análise univariada, estimando os coeficientes de correlação de Spearman.

A análise univariada foi realizada por meio da regressão de Cox para avaliar os fatores associados à mortalidade CV e geral, e os resultados foram expressos como razão de risco (HR). As seguintes variáveis foram incluídas na análise univariada: hs-TnI, sexo masculino, DM, OPG, diálise, DD, idade, Hb, TNF-alfa, IMVE, FE, PCR, fetuína, sRAGE, PTX3, FGF23, IL-6 e S100A. Posteriormente, foram realizados modelos de regressão multivariada, também utilizando Cox, incluindo variáveis clinicamente relevantes. O primeiro modelo de análise multivariada incluiu hsTnI e variáveis demográficas e clínicas. O segundo modelo utilizou as mesmas variáveis do primeiro modelo, acrescidas de parâmetros ecocardiográficos. O terceiro modelo acrescentou OPG. A medida de associação estimada foi a HR fornecida para a mudança de unidade, com um intervalo de confiança (IC) de 95%. Curvas de Kaplan-Meier foram utilizadas para análise de sobrevida. A normalidade das variáveis foi avaliada pelo teste de Shapiro-Wilk. Os valores de p < 0,05 foram considerados estatisticamente significativos. Os dados foram analisados utilizando o programa JAMOVI v. 2.5.0.

## Resultados

### Características Basais

Foram incluídos 145 pacientes (89 do sexo masculino – 54 não dialíticos, 36 em hemodiálise (HD) e 55 em diálise peritoneal). A mediana de acompanhamento foi de 36,5 meses. O número total de óbitos foi 56, e as causas específicas incluíram doenças cardiovasculares (25), infecção (17), neoplasias malignas (4) e outras causas (10). As características clínicas e bioquímicas dos pacientes, com base nos níveis de troponina e no status de sobrevida, estão resumidas nas Tabelas 1 e 2, respectivamente. A mediana do nível de hsTnI da coorte foi de 0,0703 ng/mL [0,00181; 1,68].

**Tabela 1:** Características clínicas e bioquímicas de acordo com a hsTnI, conforme definido por seu valor de corte (0,057 ng/ml) para predição de mortalidade geral e cv

	HsTnI mais elevada ≥ 0,057ng/mL n = 58	HsTnI mais baixa < 0,057ng/mL n = 87	Valor de P
Idade (anos)	62 (18;87)	58 (24;92)	0,176
Sexo (masculino)	49 (59,0%)	40 (66,7%)	0,451
Diabetes (sim)	31 (37,3%)	11 (18,3%)	0,0227
Diálise (sim)	65 (78,3%)	26 (43,3%)	0,001
Hb (g/dL)	11,2 (6;18,5)	12,5 (6,7;18,8)	0,0599
hs-CRP (mg/dL)	5,9 (0,330;74,9)	2,6 (0,3;69,1)	0,0237
IL-6 (pg/mL)	5,9 (0,330;74,9)	2,6 (0,3;69,1)	0,0546
OPG (pmol/L)	10,8 (3,72;33,2)	7,07 (1,89;18,9)	0,001
Fetuína (pg/mL)	0,418 (0,105)	0,419 (0,0984)	0,948
TNF-α (pg/mL)	18,1 (7,1;56,2)	14,5 (7,3;43,5)	0,0447
PTX3 (pg/mL)	4,12 (1,33;94,6)	3,69 (1,48;18,8)	0,32
FGF-23 (pg/mL)	507 (60;79600)	186 (44;40100)	0,15
s-RAGE (pg/mL)	2320 (540;5180)	1660 (472;5780)	0,014
S100A (ng/mL)	45,1 (5,02;464)	52,5 (6,08;160)	0,01
FE%	63 (27;84)	70 (48;88)	0,001
Disfunção diastólica	62 (74,7)	35 (58,3)	0,0593
Índice de massa do VE (g/m^2^)	62 (30,2;160)	51,5 (30,6;108)	0,00257
Troponina-I (ng/mL)	0,0322 (0,0151)	0,1811 (0,2478)	–
Morte por todas as causas	44 (53,0%)	12 (20,0%)	0,001
Morte por doença CV	25 (30,1%)	2 (3,3%)	0,001

Abreviaturas – Hb: hemoglobina; hsCRP: proteína C-reativa de alta sensibilidade; IL-6: interleucina-6; OPG: osteoprotegerina; TNF-α: fator de necrose tumoral alfa; PTX3: pentraxina 3; FGF23: fator de crescimento de fibroblastos-23; sRAGE: forma solúvel do receptor para produtos finais de glicação avançada; S100A: proteína A de ligação ao cálcio S100; FE: fração de ejeção; VE: ventrículo esquerdo; CV: cardiovascular. Nota – Variáveis quantitativas com distribuição normal são expressas como média (desvio padrão). Variáveis quantitativas com distribuição não normal são expressas como mediana (mínimo, máximo). Variáveis categóricas são expressas como frequências (porcentagem).

**Tabela 2:** Características clínicas e bioquímicas de acordo com o status de sobrevida

	Sobreviventes n = 89	Não sobreviventes n = 56	Valor de P
Idade (anos)	58 (16;83)	64 (25;92)	0,00198
Sexo (masculino)	59 (66,3%)	30 (53,6%)	0,175
Diabetes (sim)	26 (29,2%)	17 (30,4%)	1
Diálise (sim)	42 (47,2%)	49 (87,5%)	0,001
Hb (g/dL)	12,4 (7,8;18,8)	10,9 (6;18,5)	0,011
hs-CRP (mg/dL)	3,2 (0,3;54,4)	5,8 (0,660;74,9)	0,128
IL-6 (pg/mL)	3,9 (0,09;98,7)	4,9 (0,1;95,1)	0,249
OPG (pmol/L)	7,56 (1,89;18,9)	12,3 (2,37;33,2)	0,004
Fetuína (pmol/L)	0,428 (0,101)	0,407 (0,103)	0,238
TNF-α (pmol/L)	16,8 (1,5;56,2)	17,9 (7,3;34,5)	0,611
PTX3 (pg/mL)	3,83 (1,46;94,6)	4,02 (1,33;18,8)	0,17
FGF-23 (pg/mL)	240 (44;63700)	461 (61;79600)	0,69
s-RAGE (pg/mL)	1770 (540;5780)	2230 (472;5170)	0,091
S100A (ng/mL)	42,9 (6,08;464)	65,1 (5,02;409)	0,2
Troponina-I (ng/mL)	0,0521 (0,00181;0,488)	0,105 (0,0212;1,68)	0,00715
FE%	67 (31;88)	63 (27;82)	0,0654
Disfunção diastólica	55 (61,8%)	44 (78,6%)	0,0536
Índice de massa do VE (g/m^2^)	57,4 (30,2;115)	62,1 (40,6;160)	0,0826

Abreviaturas – Hb: hemoglobina; hsCRP: proteína C reativa de alta sensibilidade; IL-6: interleucina-6; OPG: osteoprotegerina; TNF-α: fator de necrose tumoral alfa; PTX3: pentraxina 3; FGF23: fator de crescimento de fibroblastos-23; sRAGE: forma solúvel do receptor para produtos finais de glicação avançada; S100A: proteína A de ligação ao cálcio S100; FE: fração de ejeção; VE: ventrículo esquerdo; CV: cardiovascular. Nota – Variáveis quantitativas com distribuição normal são expressas como média (desvio padrão). Variáveis quantitativas com distribuição não normal são expressas como mediana (mínimo, máximo). Variáveis categóricas são expressas como frequências (porcentagem).

As correlações univariadas, utilizando o coeficiente de Spearman, demonstraram que os níveis de hsTnI estiveram significativamente associados à Hb (r = -0,197; p = 0,024), PCR (r = 0,338; p < 0,001), IL-6 (r = 0,421; p < 0,001), OPG (r = 0,544; p < 0,001), TNFα (r = 0,269; p < 0,001), FE (r = -0,385, p < 0,001) e IMVE (r = 0,298; p < 0,001).

### Níveis de Troponina e Desfechos Clínicos

O valor de corte da hsTnI foi determinado com base na curva ROC para mortalidade geral e CV, sendo de 0,057 ng/mL para ambos os desfechos (AUC 0,729 [0,669–0,779]) (Figura 1). A análise univariada do risco de mortalidade revelou que níveis elevados de hsTnI (>0,057 ng/mL) estiveram relacionados a maior mortalidade geral e CV (HR 4,68; IC95%: 2,05–10,69; p < 0,001 e HR 19,18; IC95%: 2,56–143,76; p = 0.004, respectivamente), assim como os níveis de OPG (HR 5,87; IC95%: 2,83–12,21; p < 0,001 e HR 4,63; IC95%: 1,77–12,06; p = 0,002, respectivamente) e o tratamento dialítico (HR 6,31; IC95%: 2,45–16,30; p < 0,001 e HR 8,24; IC95%: 1,90–35,73; p = 0,005, respectivamente).

**Figura 1:** Curva ROC para mortalidade CV e geral entre 145 pacientes com DRC estágios 3-5. Para hsTnI de 0,057 ng/mL (valor de corte), a área sob a curva [AUC] foi de 0,729 [0,669–0,779].

As curvas de incidência cumulativa de Kaplan-Meier não demonstraram diferença na mortalidade geral (Figura 2), mas evidenciaram maior mortalidade CV (Figura 3) no grupo com hsTnI mais elevada (>0,057 ng/mL) logo no início do período de acompanhamento, com aumento da diferença ao longo do tempo. Na análise multivariada (Tabela 3), em um modelo que incluiu idade, sexo masculino, diabetes, doença CV prévia e diálise, a hsTnI foi um marcador independente de mortalidade CV. O resultado manteve-se estatisticamente significativo quando DD, IMVE e FE foram adicionados à análise. Quando a OPG foi incluída no estudo, a hsTnI permaneceu como um marcador independente de mortalidade CV (HR 12,8 (IC95% 1,56–105,08); p = 0,018). Em relação à mortalidade geral, no modelo de análise multivariada, incluindo idade, sexo masculino, diabetes, doença CV prévia e diálise, a troponina foi um marcador independente. Entretanto, quando DD, IMVE, FE e OPG foram adicionados à análise, a troponina deixou de ser um marcador independente de mortalidade geral (Tabela 4).

**Figura 2:** Curva de Kaplan Meier para mortalidade geral de acordo com os níveis basais de hsTnI em pacientes com DRC. A curva vermelha representa hsTnI < 0,057 ng/mL e a curva azul representa hsTnI ≥ 0,057 ng/mL.

**Figura 3:** Curva de Kaplan Meier para mortalidade CV de acordo com os níveis basais de hsTnI em pacientes com DRC. A curva vermelha representa hsTnI < 0,057 ng/mL e a curva azul representa hsTnI ≥ 0,057 ng/mL.

**Tabela 3:** Análise multivariada de variáveis associadas ao risco de mortalidade cv

Modelos	Variável	HR	IC 95%	Valor de p
Modelo-1	hsTnI (≥ 0,057ng/mL)	6,62	1,44–30,37	0,015
	Idade (anos)	1,01	0,97–1,05	0,621
	Sexo masculino (sim)	0,96	0,40–2,30	0,922
	DM (sim)	0,68	0,26–1,79	0,436
	DCV (sim)	1	0,37–2,76	0,995
	Diálise (sim)	4,88	1,08–22,17	0,04
Modelo-2	hsTnI (≥ 0,057ng/mL)	12,39	1,51–102	0,019
	Idade (anos)	1,01	0,97–1,06	0,565
	Sexo masculino (sim)	1,04	0,37–2,95	0,943
	DM (sim)	0,53	0,16–1,70	0,284
	Diálise (sim)	3,96	0,83–19,00	0,085
	Disfunção diastólica (sim)	0,92	0,33–2,58	0,873
	DCV (sim)	0,76	0,22–2,68	0,674
	FE (%)	1	0,95–1,04	0,897
	Índice de massa do VE (g/m^2^)	1,01	0,99–1,04	0,342
Modelo-3	hsTnI (≥ 0,057 ng/mL)	12,8	1,56–105,08	0,018
	Idade (anos)	1,02	0,97–1,07	0,490
	Sexo masculino (sim)	1,09	0,38–3,16	0,847
	DM (sim)	0,53	0,16–1,70	0,284
	Diálise (sim)	4,30	0,77–23,90	0,097
	Disfunção diastólica (sim)	0,94	0,31–2,81	0,912
	FE (%)	1,01	0,95–1,04	0,789
	Índice de massa do VE (g/m^2^)	1,01	0,99–1,04	0,290
	OPG (pmol/L)	0,99	0,91–1,09	0,881
	DCV (sim)	0,86	0,23–3,21	0,817

Abreviaturas – OPG: osteoprotegerina; DCV: doença cardiovascular; DRC: doença renal crônica; hsTnI: troponina I de alta sensibilidade; HR: razão de risco; IC95%: intervalo de confiança de 95%.

**Tabela 4:** Análise multivariada de variáveis associadas ao risco de mortalidade por todas as causas

Modelos	Variável	HR	IC 95%	Valor de p
Modelo-1	hsTnI (≥ 0,057ng/ml)	2,15	1,02–4,54	0,044
	Idade (anos)	1,03	1,01–1,05	0,046
	Sexo masculino (sim)	0,64	0,36–1,15	0,135
	DCV (sim)	0,97	0,48–1,99	0,944
	DM (sim)	0,81	0,41–1,57	0,529
	Diálise (sim)	4,16	1,76–9,83	0,001
Modelo-2	hsTnI (≥ 0,057 ng/mL)	1,97	0,81–4,77	0,133
	Idade (anos)	1,03	1,00–1,06	0,098
	Sexo masculino (sim)	0,58	0,30–1,14	0,115
	DM (sim)	0,77	0,37–1,61	0,489
	DCV (sim)	0,78	0,35–1,75	0,544
	Diálise (sim)	4,11	1,65–10,20	0,002
	Disfunção diastólica (sim)	1,42	0,62–3,25	0,413
	FE (%)	0,95	0,93–1,01	0,247
	Índice de massa do VE (g/m^²^)	1,01	0,99–1,02	0,533
Modelo-3	hsTnI (≥ 0,057 ng/mL)	1,96	0,81–4,77	0,138
	Idade (anos)	1,03	0,99–1,06	0,124
	Sexo masculino (sim)	0,58	0,29–1,14	0,114
	DM (sim)	0,77	0,37–1,61	0,489
	Diálise (sim)	4,01	1,44–11,16	0,008
	Disfunção diastólica (sim)	1,40	0,58–3,38	0,460
	Índice de massa do VE (g/m^2^)	1,01	0,99–1,02	0,545
	FE (%)	0,98	0,95–1,01	0,245
	OPG (pmol/L)	1,00	0,94–1,07	0,928
	DCV (sim)	0,78	0,34–1,77	0,556

Abreviaturas – OOPG: osteoprotegerina; DCV: doença cardiovascular; DRC: doença renal crônica; hsTnI: troponina I de alta sensibilidade; HR: razão de risco; IC de 95%: intervalo de confiança de 95%.

## Discussão

A hsTnI demonstrou ser um marcador independente de mortalidade CV em pacientes com DRC, independentemente de sexo, idade, marcadores inflamatórios e oxidativos e outros fatores de risco CV estabelecidos, incluindo parâmetros ecocardiográficos e OPG. O mecanismo exato que explica a elevação da troponina e sua associação com a mortalidade ainda é desconhecido^11^. A hipótese é que doença arterial coronariana subclínica, desequilíbrio entre oferta e demanda relacionado à hipertrofia cardíaca ou injúria miocárdica direta possam desempenhar um papel nesse processo^12^.

Nossos achados confirmam uma maior mortalidade CV em pacientes com DRC com níveis aumentados de hsTnI, independentemente da terapia dialítica, demonstrando que a hsTnI pode ser um marcador independente de mortalidade CV nessa população. A hsTnI pode ser mais específica do que a TnT para indicar doença cardíaca subjacente e, consequentemente, risco de mortalidade CV^13^. Em uma coorte de 20 mil indivíduos que comparou medições de hsTnI e hsTnT e desfechos clínicos, Welsh et al.^9^ descobriram que ambos os marcadores apresentaram associações semelhantes e robustas com morte por causas CV, mas, após o ajuste para fatores de risco estabelecidos, apenas a TnI foi associada a infarto do miocárdio e doença coronariana crônica. De forma semelhante ao nosso estudo, a TnI não apresentou associação com óbito não relacionado a DCV. Esse achado também está de acordo com estudos anteriores que sugeriram que a TnT foi mais influenciada por doenças não cardíacas^14,15^.

Apesar de diversos estudos relatarem a associação da troponina com a mortalidade CV, ainda existe incerteza. Bargnoux constatou que a PCR e o peptídeo natriurético cerebral foram preditores independentes de mortalidade em pacientes em HD, em contraste com a TnI, que não apresentou associação com a mortalidade geral^16^. Satyan demonstrou um achado semelhante com relação à TnT^17^. Nosso estudo não encontrou uma associação entre PCR e mortalidade CV ou geral, seja na análise univariada ou multivariada, mas encontrou uma associação positiva entre hsTnI e mortalidade CV, o que pode ser explicado pelo ensaio de alta sensibilidade. Em contraste com nossos achados, um estudo anterior de nosso grupo identificou uma associação positiva entre marcadores inflamatórios e mortalidade CV em pacientes com DRC^18^. Um estudo de coorte sobre marcadores de mortalidade CV na DRC não encontrou associações com PCR e mortalidade CV nos estágios iniciais da DRC e postulou que a inflamação poderia mediar a mortalidade apenas em estágios avançados^19^. Uma vez que incluímos pacientes em diálise e não dialíticos, isso pode explicar por que marcadores inflamatórios não foram associados à mortalidade.

Nosso estudo está em conformidade com uma metanálise que avaliou o impacto prognóstico da TnT e da TnI em pacientes com DRC; no entanto, poucos dos estudos incluídos realizaram análise multivariada com dados ajustados ou utilizaram ensaios de troponina de alta sensibilidade^7^. Isso destaca a relevância de nosso estudo, que aplicou a análise da curva ROC para determinar um valor de corte de troponina capaz de predizer a mortalidade CV e ajustado para variáveis, incluindo OPG, um marcador de calcificação vascular, inflamação e um forte preditor de mortalidade na DRC^20^.

Pacientes com DRC podem apresentar ecocardiografia normal e, ainda assim, exibir aumento da morbimortalidade CV^21^. No entanto, a remodelação cardíaca é comum e pode determinar um pior prognóstico^22^. Hipertrofia do ventrículo esquerdo, FE reduzida e DD são alguns dos sinais ecocardiográficos classicamente relacionados a um prognóstico desfavorável tanto na população geral quanto em pacientes com DRC^23, 24, 25, 26^. Constatamos que o aumento do IMVE e a redução da FE foram mais frequentes em pacientes com níveis mais elevados de troponina, o que está em consonância com a literatura^27^.

Embora as alterações ecocardiográficas tenham sido associadas ao aumento da mortalidade na DRC, estudos anteriores não incluíram a hsTnI na análise multivariada^28, 29, 30, 31^. Sun et al.^27^ avaliaram a associação da hsTnT com desfechos CV em pacientes em HD e constataram que DD e IMVE não apresentaram associação com mortalidade CV e geral quando ajustados para hsTnT. Demonstramos achados semelhantes, uma vez que a hsTnI permaneceu como um marcador independente de mortalidade CV, independentemente da FE, do IMVE e da DD.

O presente estudo tem diversas limitações que devem ser ressaltadas. Em primeiro lugar, seu desenho observacional restringe as inferências causais entre os níveis de hsTnI e a mortalidade cardiovascular. Em segundo lugar, a análise da troponina foi baseada em uma única amostra, o que limita a avaliação de alterações dinâmicas e suas potenciais implicações prognósticas. Terceiro, embora o tamanho geral da amostra tenha sido adequado para as análises primárias, ele limitou o poder de avaliações detalhadas de subgrupos, particularmente entre pacientes em diálise e pacientes não dialíticos e indivíduos diabéticos e não diabéticos. Estudos futuros com amostras maiores e medições seriadas de biomarcadores são necessários para validar e expandir esses achados.

Em resumo, o presente estudo revelou que a hsTnI é um forte indicador prognóstico de mortalidade CV em pacientes com DRC, enquanto sua associação com a mortalidade geral foi fraca. Esses achados sugerem que a TnI tem um papel potencial na estratificação do risco CV na população com DRC. O valor de corte identificado (0,057 ng/mL) pode constituir uma ferramenta clínica valiosa para identificar pacientes de alto risco que possam se beneficiar de um monitoramento intensificado e intervenções terapêuticas precoces com o objetivo de reduzir eventos CV. A incorporação da medição de hsTnI na prática nefrológica de rotina pode facilitar a avaliação personalizada do risco, orientando os médicos na definição de estratégias de manejo individualizadas, como o controle mais agressivo dos fatores de risco tradicionais e o encaminhamento imediato para a cardiologia. Ainda assim, são necessários mais estudos para melhor compreender os mecanismos relacionados à elevação da troponina e sua associação com a mortalidade, para avaliar o desempenho da hsTnI como parte de modelos prognósticos de risco em combinação com outros biomarcadores e, por fim, para investigar possíveis terapias para reduzir o risco CV demonstrado.

## Data Availability

Todos os dados que sustentam os achados deste estudo estão disponíveis com a autora correspon­dente, Gabriela Romaniello, por e-mail (romaniello.gabriela@gmail.com), mediante solicitação razoável. O conjunto de dados não está disponível publicamente devido à presença de informações de saúde pessoalmente identificáveis ou sensíveis, o que poderia comprometer a confidencialidade dos participantes.
